# Diacerein ameliorates cholestasis-induced liver fibrosis in rat via modulating HMGB1/RAGE/NF-κB/JNK pathway and endoplasmic reticulum stress

**DOI:** 10.1038/s41598-023-38375-4

**Published:** 2023-07-15

**Authors:** Amira Mohammed Abdelfattah, Shireen Sami Mahmoud, Dalia Ibrahim EL-wafaey, Heba Mahmoud Abdelgeleel, Amira Mohamed Abdelhamid

**Affiliations:** 1 Clinical Pharmacology Department, Faculty of Medicine, Zagazig University, Zagazig, Sharkia Egypt; 2 Human Anatomy and Embryology Department, Faculty of Medicine, Zagazig University, Zagazig, Egypt; 3 Pathology Department, Faculty of Medicine, Zagazig University, Zagazig, Sharkia Egypt

**Keywords:** Gastroenterology, Molecular medicine

## Abstract

Diacerein is an interleukin (IL)-1β inhibitor approved for osteoarthritis. This study aimed to investigate the potential anti-fibrotic effect of diacerein against bile duct ligation (BDL)-induced liver fibrosis. Forty male Wistar rats were divided into: sham-operated group, BDL group, and BDL groups treated with diacerein at 10, 30, and 50 mg/kg/day starting two days before surgery and continued for 4 weeks. Diacerein decreased the hepatic injury markers and alleviated oxidative stress triggered by BDL by reducing hepatic malondialdehyde (MDA) and increasing hepatic superoxide dismutase (SOD) levels. Diacerein mitigated BDL-induced inflammation via lowering hepatic levels and mRNA expression of high mobility group box 1 (HMGB1), nuclear factor-κB (NF-κB), and IL-1β. The hepatic gene expression of Advanced Glycation End products Receptor (RAGE) gene and immunohistochemical expression of some ER stress markers, e.g., glucose-regulated protein 78 (GRP78), inositol-requiring enzyme 1 (IRE1α), protein kinase RNA-like endoplasmic reticulum kinase (PERK), CCAAT/enhancer-binding protein homologous protein (CHOP), and phosphorylated c-Jun N-terminal kinase protein contents were lowered by diacerein. Furthermore, diacerein suppressed the hepatic levels of fibrogenic mediators, e.g., Transforming growth factor β1 (TGF˗β1), α- smooth muscle actin (α-SMA), collagen 1, and hydroxyproline, as well as the apoptotic caspase 3 and BAX immunostaining in BDL rats. The histopathological abnormalities induced by BDL significantly improved. Our study demonstrated that diacerein exhibited an antifibrotic effect by inhibiting HMGB1/RAGE/NF-κB/JNK pathway, and ER stress. Better protection was observed with increasing the dose.

## Introduction

Cholestatic liver disease is an important morbid condition initiated by impairment of bile formation and/or flow^1^. Stagnation of harmful hydrophobic bile salts resulting from obstructive cholestasis elicits deleterious effects on different liver cells, causing free radical generation, inflammatory burdens, apoptosis, fibrosis, and consequent cirrhosis^2^. Bile salt stagnation within the liver generates a ductular response in which biliary epithelial cells and intrahepatic bile ducts multiply. This response is accompanied by Kupffer cell activation and the infiltration of many inflammatory cells, which generate a plethora of pro-inflammatory chemicals that activate hepatic stellate cells (HSCs) with the subsequent generation of transforming growth factor β (TGF-β). All these changes result in an increase in the number of α-smooth muscle actin (α-SMA) positive cells derived from dormant HSCs, which produce a massive amount of extracellular matrix proteins (ECM) that is deposited in the hepatic parenchyma, inducing fibrosis^3^.

Liver fibrosis is a complex pathological process^4^. Inflammation is considered a key driving factor in the fibrogenesis, which promotes the transdifferentiation of HSCs into myofibroblasts with the subsequent generation of fibrogenic cytokines, and ECM. Accordingly, inhibiting the inflammatory mediators may be an effective treatment option for cholestasis-induced liver fibrosis^5^.

High-mobility group box 1 (HMGB1) is a nuclear non-histone protein that has been known as damage-associated molecular patterns (DAMPs)^6^. HMGB1 is expelled either passively or actively from cells under pathological conditions, where it reacts with its cell surface receptors, eliciting numerous intracellular cascades^7^. It is considered an important pro-inflammatory cytokine^8^. It is actively involved in the fibrotic remodelling of different organs, such as myocardial, pulmonary, cystic, and renal fibrosis^9^. In addition, HMGB1 was notably elevated in rat fibrotic liver induced by CCl_4_^10^, and in patients and mice with schistosomiasis liver fibrosis^11^.

Receptor for advanced glycation end products (RAGE) is a member of the antibody superfamily of receptors abundantly expressed on different types of liver cells, e.g., hepatocytes, Kupffer cells, and HSCs and has a strong binding affinity for HMGB1^12^. The HMGB1/RAGE complex may play a key role in fibrosis. Wang et al.^13^ illustrated that the HMGB1/RAGE interaction mediates lung inflammation and fibrosis in a rat lung injury induced by arsenic. Based on these data, inhibiting extracellular HMGB1 activity or preventing its release may be beneficial therapeutic targets in treating cholestasis-induced liver fibrosis.

Endoplasmic reticulum (ER) stress is a phenomenon generated by a broad range of stressful stimuli^14^. The accumulated unfolded proteins trigger ER stress sensor proteins, including inositol-requiring enzyme 1 (IRE1α), activating transcription factor 6 (ATF6), and protein kinase RNA-like endoplasmic reticulum kinase (PERK), with subsequent effects on numerous intracellular pathways^15^. Emerging studies reported that ER stress is involved in diverse fibrotic disorders, such as lung fibrosis and chronic liver disease with fibrosis^16,17^. Prior studies have found that HMGB1 is the upstream regulator of ER stress^18,19^.

Diacerein, an anti-inflammatory medication of the anthraquinone derivative class, has been approved for treatment of osteoarthritis^20^. Its primary molecular mechanism of action is inhibiting IL˗1β and its downstream signalling^21^. Additionally, diacerein reduces the synthesis and activity of numerous cytokines, nitric oxide, and neutrophils' phagocytic activity^22^. Moreover, diacerein also prevents both IκB-α degradation and NF-κB activation^23^. Prior experimental and clinical studies have established the anti-inflammatory effects of diacerein^24,25^.

To our knowledge, there is no particular evidence demonstrating protective effect of diacerein against cholestasis-induced liver fibrosis in rats. Therefore, the current study goal was to elucidate the potential anti-fibrotic effects of diacerein on BDL-induced liver fibrosis and the probable related mechanism, with emphasis on HMGB1/RAGE pathway.

## Materials and methods

### Drugs and chemicals

Diacerein powder and sodium carboxymethyl cellulose [CMC-Na] were purchased from Sigma Aldrich, Cairo, Egypt. Thiopental sodium was obtained from Sigmatec Pharmaceutical Industries, Egypt. Other chemicals were commercially available.

### Animals

Forty adult male Wistar rats (250–300 gm/rat, 8–10 weeks old) were purchased from the animal house of the Faculty of Veterinary Medicine, Zagazig University, Egypt. Rats were housed under standard conditions of temperature (28 ± 2 °C), humidity, and 12-h light/dark cycles and were left for acclimatization for one week prior to experimentation. Standard laboratory diet and water were allowed ad libitum.

### Ethics statement

This experimental study design was accepted by the Institutional Animal Care and Use Committee *(IACUC)* at Zagazig University, Faculty of Medicine, Egypt on 29th December 2020 (approval no. ZU–IACUC/3/F/144/2020). All the procedures followed the international ethical guidelines for the care and use of laboratory animals, and complied with the ARRIVE guidelines.

### Experimental design

Rats were randomly allocated to five groups (n = 8 in each) as follows: Group I (sham-operated group), Group II (bile duct ligated [BDL] group): rats in both groups received 1 ml of 0.5% CMC (vehicle of diacerein), Group III, IV, and V (BDL + Diacerein groups): rats were exposed to BDL and administered 10, 30, and 50 mg/kg diacerein, respectively. Rats received diacerein and CMC orally once daily for four weeks, starting two days before surgery.

The selection of doses of diacerein, 30 and 50 mg/kg/day, was based on a previous study demonstrating its tissue protective effect^26^, while the dose of diacerein, 10 mg/kg/day, was selected by converting the human dose of diacerein (50 mg twice/day) using the pharmacokinetic formula for humans and rats^27^.

The induction of cholestatic liver fibrosis was made through the common bile duct ligation. Under aseptic conditions, rats were anesthetized with thiopental sodium 50 mg/kg given by intraperitoneal (i.p.) injection, then a 1.5 cm median laparotomy was made, and the common bile duct was isolated from the surrounding tissue and ligated at two points with 4.0 silk ligatures. Sham-operated rats were subjected to the same midline incision and manipulation of the common bile duct, but without ligation^28^.

### Collection of blood and liver tissue samples

At the end of the experiment, rats were anesthetized with thiopental (50 mg/kg i.p.), then blood samples were collected from the retro-orbital plexus in heparinized tubes and centrifuged at 3000 rpm for 15 min. The serum samples were aspirated and stored at – 20 °C until examined.

All rats were sacrificed immediately after blood sampling by decapitation, then a median laparotomy was made, and the liver from each rat was dissected, immediately removed, and divided into two parts: one part was snap frozen in liquid nitrogen and stored at − 80 °C to be used for biochemical and gene expression studies, whereas the other part was used for histopathological and immunohistochemical analysis after fixation in 10% neutral formalin.

### Biochemical analysis

Serum levels of alanine aminotransferase (ALT) and aspartate aminotransferase (AST) were measured spectrophotometrically using commercially available kits provided by Spectrum Diagnostics (Cairo, Egypt). Additionally, serum levels of alkaline phosphatase (ALP) and direct bilirubin (DB) were assayed using kits purchased from Biodiagnostics (Giza, Egypt). Malondialdehyde (MDA) contents, as a lipid peroxidation indicator, and superoxide dismutase (SOD), as an enzymatic antioxidant biomarker, were measured in liver tissue homogenates using commercially available colorimetric assay kits provided by Biodiagnostic (Giza, Egypt). All procedures were conducted as per manufacturers’ instructions.

### Enzyme-linked immunosorbent assay (ELISA)

The quantitative sandwich ELISA technique was used for estimating the hepatic concentrations of high-mobility group box 1 (HMGB1), nuclear factor-κB (NF-κB), interleukin-1 beta (IL-1β), transforming growth factor β1 (TGF-β1), hydroxyproline, and caspase 3 using ELISA kits from MyBioSource (San Diego, CA, USA). The p-JNK ELISA kits purchased from RayBiotech (Life, Georgia, USA). Moreover, collagen type 1 (COL-1) and α-smooth muscle actin (α-SMA) in hepatic homogenate were also assayed using ELISA kits (CUSABIO, USA). The analysis was carried out according to the manufacturers’ protocol.

### Quantitative real time-polymerase chain reaction (RT-PCR)

Rats’ liver tissue samples were homogenized, and total RNA was extracted using Trizol (Thermo Fisher Scientific, Waltham, MA, USA). The quality and quantity of the extracted RNA was assessed via Spectrophotometer by evaluating the OD at 260 and 280 nm and accepting A260/A280 at a ratio of 1.8–2.1. RNA was reversely transcribed to cDNA using a Revert Aid First Strand cDNA Synthesis kit (Fermentas, Vilnius, Lithuania). The cDNA was subjected to quantitative RT-PCR implemented in a StepOnePlus Real-Time PCR system (Applied Biosystems, USA) using iQ SYBR Green supermix (BioRad Laboratories, Hercules, CA, USA). The primers’ sequences (Thermo Fischer Scientific, USA) were as follows:

**Table:** 

Gene	Species	Primers	Product length	Accession number
HMGB-1	Rat	Forward: 5′-AGGCTG ACAAGGCTCGTTATG-3′ Reverse: 5′-TGTCATCCGCAGCAG TGTTG-3′	228	NM_012963.3
RAGE	Rat	Forward: 5′-CTGCCTCTGAACTCACAGCCAATG-3′ Reverse: 5′-GTGCCTCCTGGTCTCCTCCTTC-3′	155	NM_053336.2
GRP78	Rat	Forward: 5′-AAGAGCCAGGATTCTCAGCG-3′ Reverse: 5′-GGGGTCTTGTCGTTGTCAGT-3′	238	M14866.1
IRE1α	Rat	Forward: 5′-GAGGAATTACTGGCTTCTCATAGG‐3′ Reverse: 5′-TTCTCGATGTTTGGGAAGATTG‐3′	100	NM_001191926.1
PERK	Rat	Forward: 5′-CTGCAATCATCCGTCAGGGT-3′ Reverse: 5′-GCTTCCATTTGATCGTCGGC-3′	109	NM_031599.2
Rplp1	Rat	Forward: 5′-TAAGGCCGCGTTGAGGTG-3′ Reverse: 5′-GATCTTATCCTCCGTGACCGT-3′	150	NM_001007604.2

HMGB-1 (High Mobility Group Box 1), RAGE (Advanced Glycation End products Receptor), GRP78(glucose-regulated protein 78), IRE1α (Inositol-requiring enzyme 1), PERK (PKR-like ER protein kinase), Rplp1 (Ribosomal protein lateral stalk subunit P1).

The level of expression of each gene was normalized to the level of the housekeeping gene Rplp1. The results were detected as fold changes by the 2 –ΔΔCT method by Livak and Schmittgen^29^.

### Histopathological examinations

Liver specimens were fixed in 10% buffered formalin solution for 24–48 h, dehydrated in increasing grades of ethanol, and then embedded in paraffin wax. sections 3–4 μm thick were cut and subjected to the following: hematoxylin and eosin (H&E) stain for histological evaluation and Masson’s trichrome stain to detect collagen fibers.

Consistent with the method described by Batts and Ludwig^30^, the grade of inflammation and stage of hepatic fibrosis were evaluated in individual biopsy specimens. The severity of inflammation was graded on a scale of 0 to 4 [0: no activity; 1: minimal; 2: mild; 3: moderate; and 4: severe]. The fibrosis score was evaluated on a five-point scale [F0: no fibrosis; F1: portal fibrosis without septa; F2: peri-portal fibrosis; F3: septal fibrosis without cirrhosis; and F4: definite cirrhosis].

Histomorphometry analysis of hepatic sections stained with Masson’s trichrome by Image J analysis software (Fiji Image J; 1.51 n, NIH, USA) was done at the Human Anatomy and Embryology Department, Faculty of Medicine, Zagazig University, and the area percent of collagen fibers was evaluated and measured in five non-overlapping histological fields/sections from each rat in the study groups.

### Immunohistochemical evaluation of CHOP and BAX

For CHOP and BAX immunostaining, the slices on charged slides were placed in an EDTA pH 9.0 buffer and microwaved for antigen retrieval with heat. The slices were washed with PBS three times for 5 min each after natural cooling, followed by probing with mouse anti-CHOP antibodies (Santa Cruz, United States; 1:50) and rabbit anti-Bax antibodies (E63, 1:250, Abcam, UK). CHOP and BAX were assessed by brown cytoplasmic staining. The intensity was scored from 0 to 3 based on the method described by Allred et al.^31^, as 0: negative; 1: detectable but weak; 2: moderate; and 3: strong at high power fields (X400) per liver section.

### Statistical analysis

All values were expressed as means ± standard error (SE). Group means were compared by one-way analysis of variance (ANOVA) with post hoc Tukey test for pairwise comparisons. In addition, Chi-square test was used for the histological contingency table. The tests were carried out using GraphPad Prism version 5.00 (GraphPad Software, San Diego, California; USA). Values of p < 0.05 were considered statistically significant, while values of p < 0.001 were highly statistically significant.

## Results

### Effect of diacerein on serum liver transaminases

**Figure 1:**
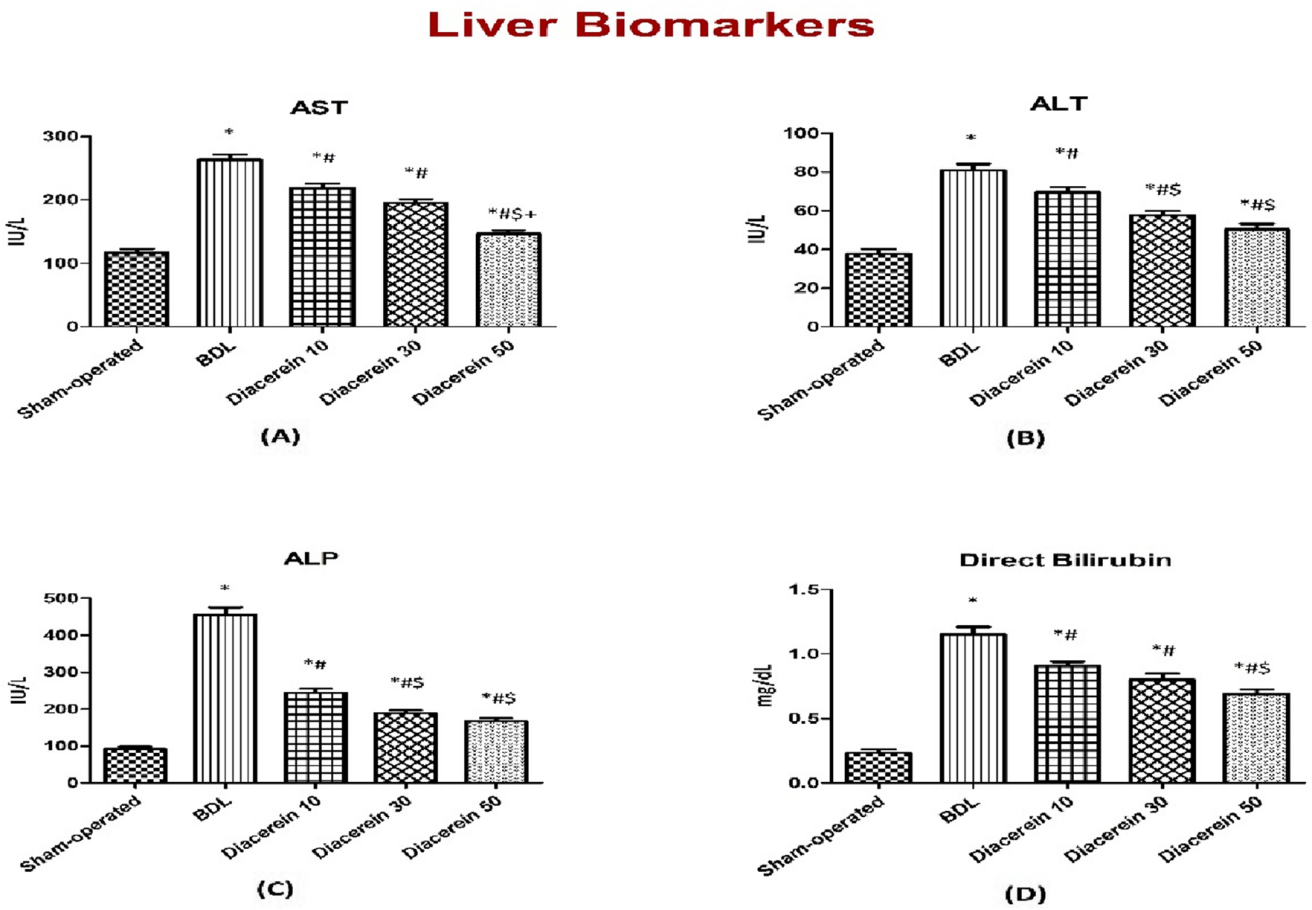
Bar charts showing the effect of oral administration of different doses of diacerein on liver biomarkers in different experimental groups. (**A**) Serum AST level (IU/L) (**B**) Serum ALT level (IU/L) (**C**) Serum ALP level (IU/L) (**D**) Serum direct bilirubin level (mg/dL). The values of different groups were presented as means ± SE (n = 8 rats per group). *p < 0.05 versus the sham-operated group; ^#^p < 0.05 versus the BDL group; ^$^p < 0.05 versus the diacerein-treated group (10 mg/kg/day); ^+^p < 0.05 versus the diacerein-treated group (30 mg/kg/day).

Serum levels of AST and ALT in the BDL group were significantly (p < 0.05) higher than those of the sham-operated group. Treatment of rats with diacerein at doses of 10, 30, and 50 mg/kg/day for four weeks showed a significant (p < 0.05) decrease in the serum levels of AST and ALT compared to the BDL group yet were still significantly (p < 0.05) higher as compared to the sham-operated group. The reduction in liver transaminases was greater with higher doses of diacerein (Fig. 1A,B).

### Effect of diacerein on serum cholestatic indices

Rats exposed to BDL demonstrated a significant (p < 0.05) elevation in serum levels of ALP and direct bilirubin compared to the sham-operated rats. Meanwhile, rats that received diacerein at doses of 10, 30, and 50 mg/kg/day for 4 weeks showed a significant (p < 0.05) reduction of these parameters in a dose-dependent manner compared to the BDL rats yet were still significantly (p < 0.05) higher as compared to the sham-operated group (Fig. 1C,D).

### Effect of diacerein on oxidant/antioxidant parameters in liver tissue

**Figure 2:**
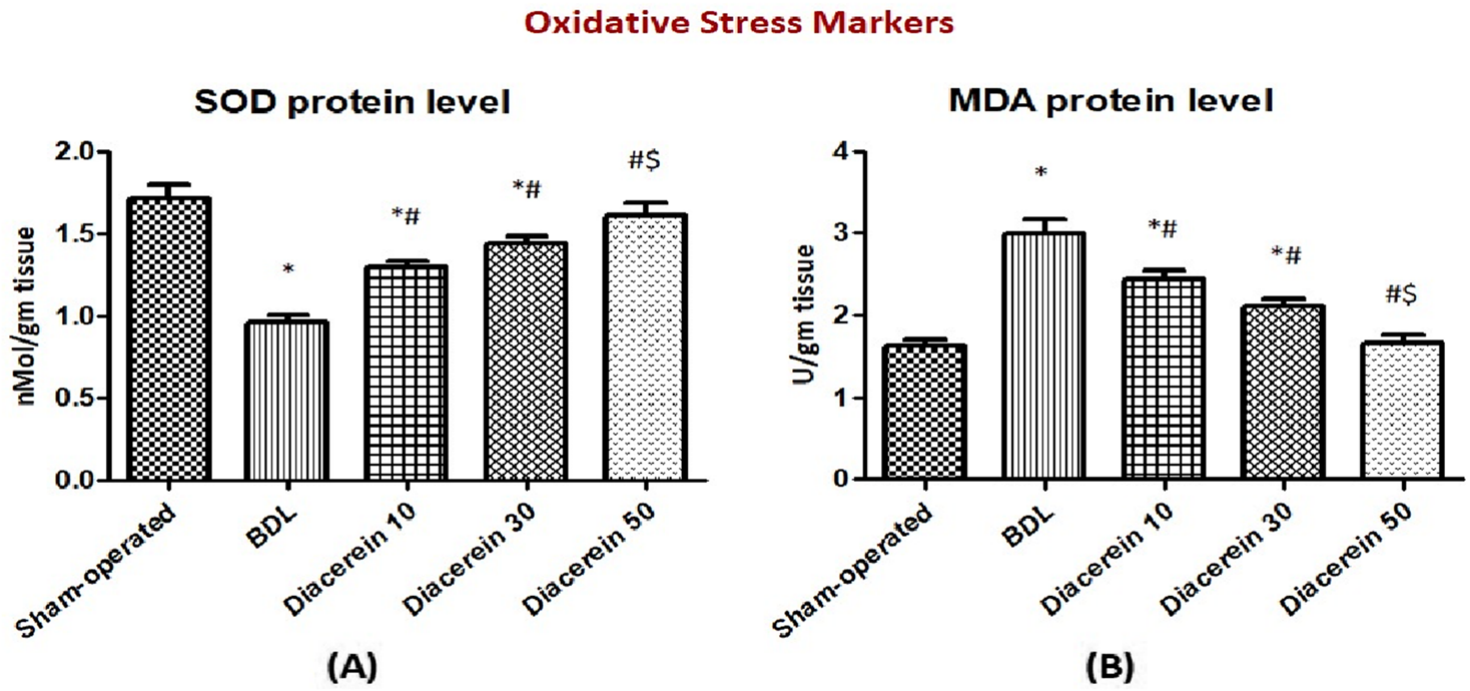
Bar charts showing the effect of oral administration of different doses of diacerein on hepatic levels of (**A**) SOD (nMol/gm tissue) and (**B**) MDA (U/gm tissue) in different experimental groups. The values of different groups were presented as means ± SE (n = 8 rats per group). *p < 0.05 versus the sham-operated group; ^#^p < 0.05 versus the BDL group; ^$^p < 0.05 versus the diacerein-treated group (10 mg/kg/day); ^+^p < 0.05 versus the diacerein-treated group (30 mg/kg/day).

A significant (p < 0.05) increase in hepatic MDA levels accompanied by a significant (p < 0.05) decrease in hepatic SOD levels were demonstrated in rats exposed to BDL as compared to those of the sham-operated rats. On the other hand, a significant (p < 0.05) decrease in hepatic MDA levels and a significant (p < 0.05) elevation of hepatic SOD levels were observed in rats treated with diacerein in comparison to the BDL group. This effect of diacerein was dose-related (Fig. 2A,B).

### Effect of diacerein on hepatic levels and gene expression of HMGB1 and RAGE

**Figure 3:**
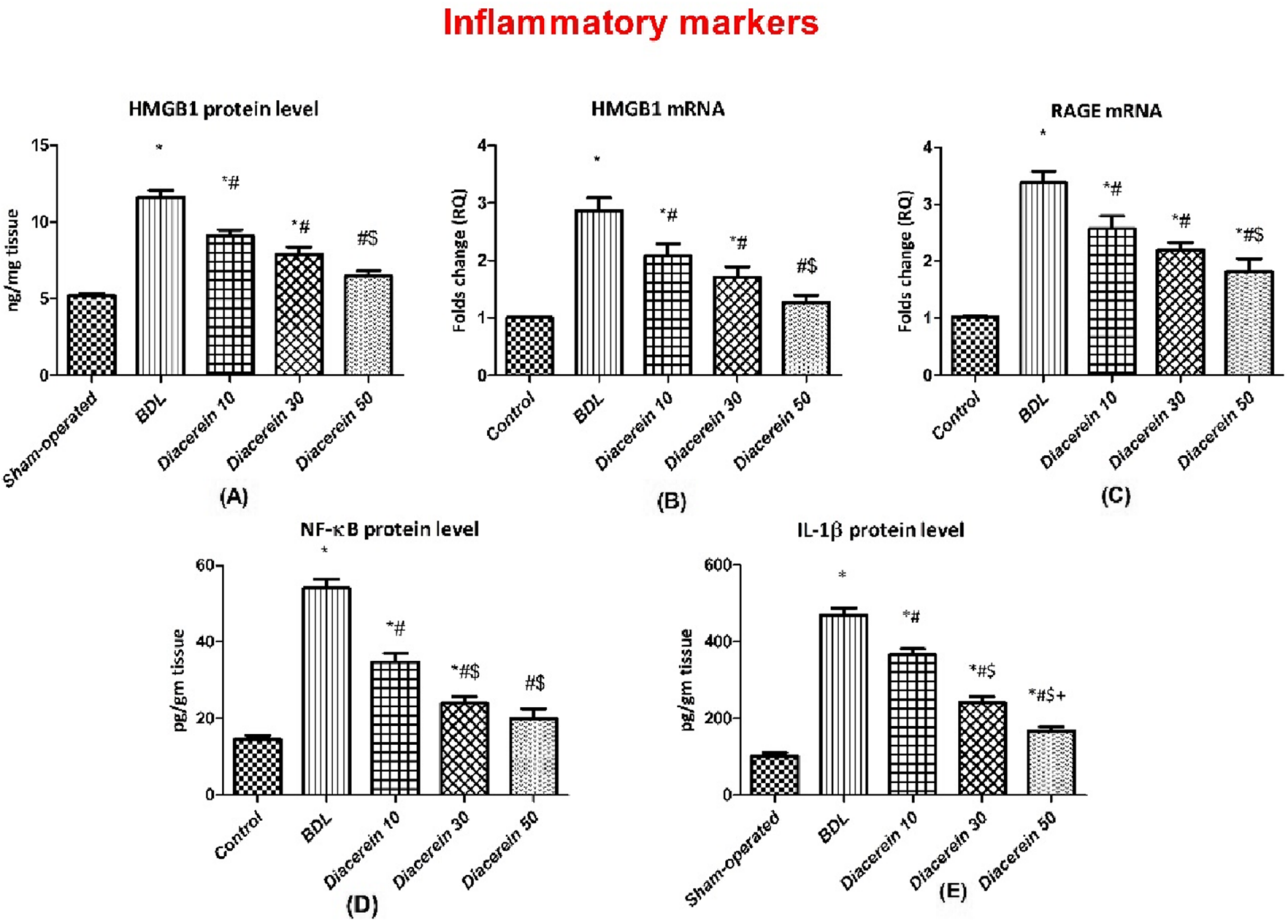
Bar charts showing the effect of oral administration of different doses of diacerein on hepatic levels of (**A**) HMGB1 protein content (ng/mg tissue), (**B**) HMGB1 gene expression (folds change), (**C**) RAGE gene expression (folds change), (**D**) NF-κB protein content (pg/gm tissue), and (**E**) IL-1β protein content *(pg/gm tissue)* in different experimental groups. The values of different groups were presented as means ± SE (n = 8 rats per group). *p < 0.05 versus the sham-operated group; ^#^p < 0.05 versus the BDL group; ^$^p < 0.05 versus the diacerein-treated group (10 mg/kg/day); ^+^p < 0.05 versus the diacerein-treated group (30 mg/kg/day).

Rats exposed to BDL showed a significant (p < 0.05) elevation in the hepatic protein level of HMGB1 as well as the mRNA expression of HMGB1 and RAGE as compared to the sham-operated rats. While treatment of rats with diacerein significantly (p < 0.05) lowered, in a dose-dependent manner, the HMGB1 hepatic protein level and the relative expression of both genes as compared to the BDL rats (Fig. 3A–C).

### Effect of diacerein on hepatic NF-κB and IL˗1β levels

Hepatic NF-κB and IL˗1β levels in BDL rats were significantly (p < 0.05) higher than those of the sham-operated rats. However, daily administration of diacerein for four weeks significantly (p < 0.05) decreased the levels of NF-κB and IL˗1β, with better improvement with increasing the dose (Fig. 3D,E).

### Effect of diacerein on the ER stress signalling pathway (GRP78/IRE1α/PERK/CHOP)

**Figure 4:**
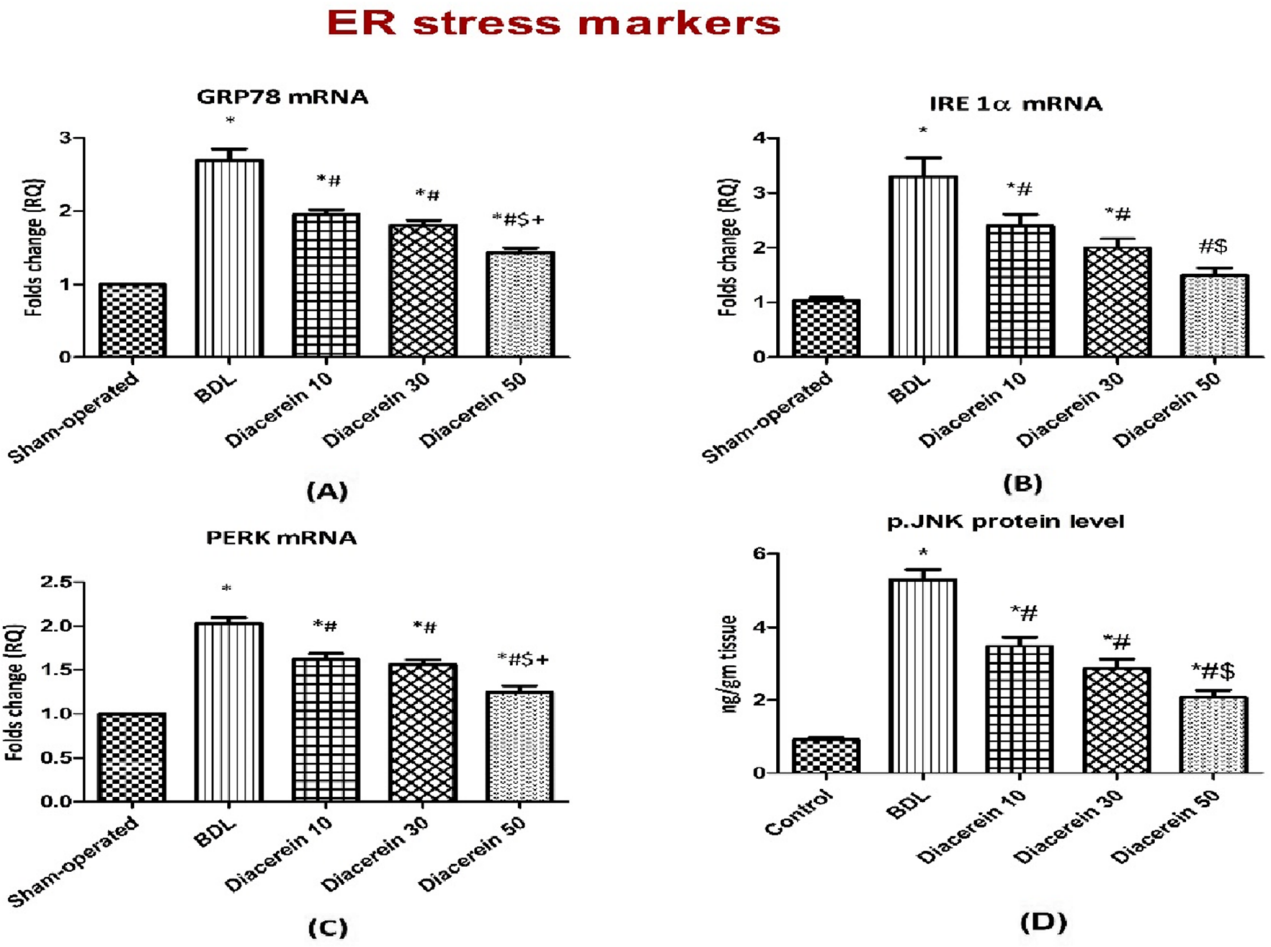
Bar charts showing the effect of oral administration of different doses of diacerein on ER stress-related parameters: (**A**) GRP78 gene expression (folds change), (**B**) IRE 1α gene expression (folds change), (**C**) PERK gene expression (folds change), and (**D**) p-JNK protein content (ng/g tissue) in liver tissue of different experimental groups. The values of different groups were presented as means ± SE (n = 8 rats per group). *p < 0.05 versus the sham-operated group; ^#^p < 0.05 versus the BDL group; ^$^p < 0.05 versus the diacerein-treated group (10 mg/kg/day); ^+^p < 0.05 versus the diacerein-treated group (30 mg/kg/day).

BDL-induced ER stress in the liver tissue homogenates was evidenced by significant (p < 0.05) up-regulation in GRP78, IRE1α, and PERK *mRNA* expression when compared to the sham-operated rats. Treatment of rats with 10, 30, and 50 mg/kg/day diacerein showed significant (p < 0.05) down-regulation in the expression of these genes in the liver tissue (Fig. 4A–C).

**Figure 5:**
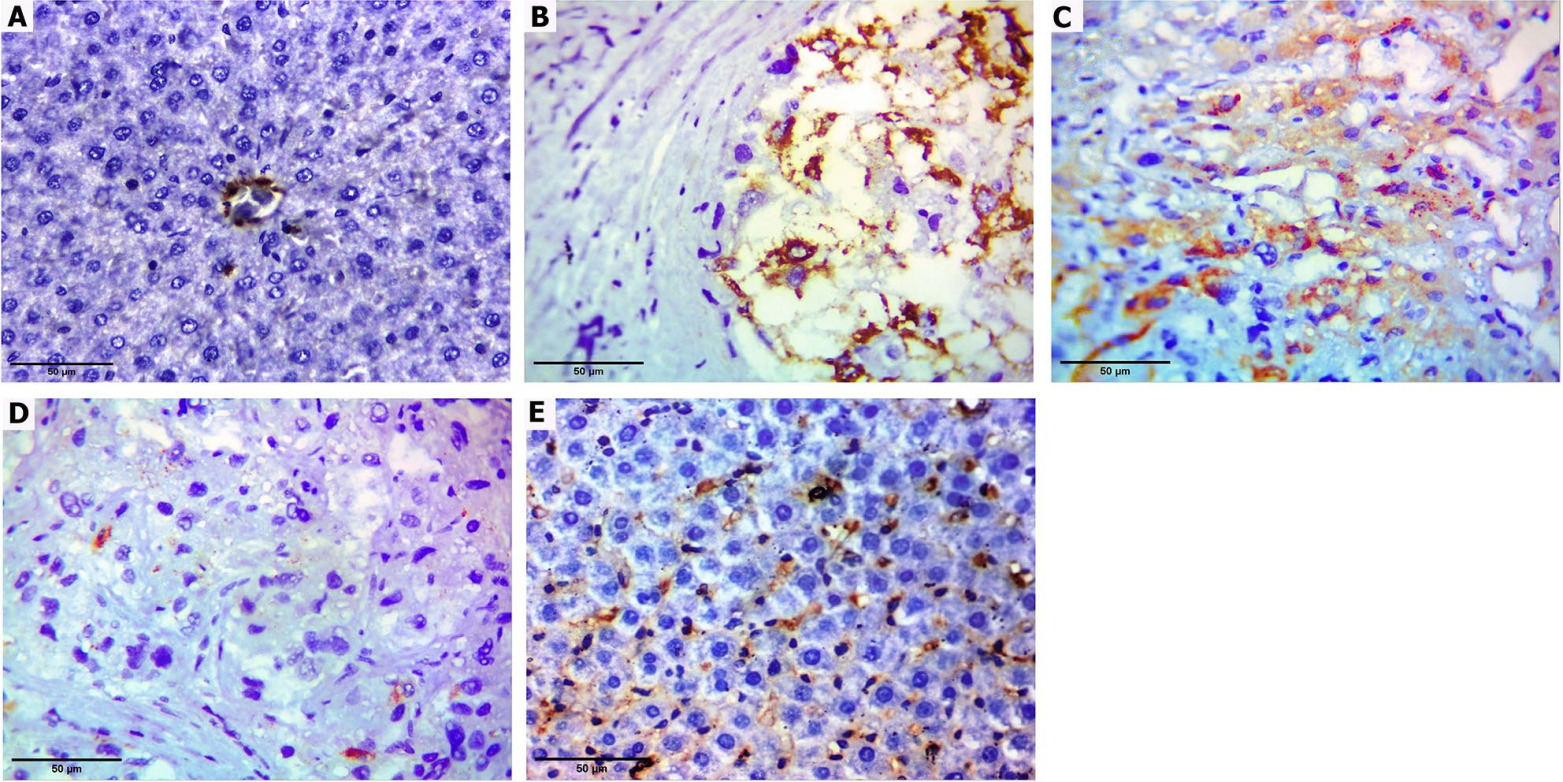
Immunohistochemistry staining images X400 showing hepatic expression of CHOP (**A**) Sham-operated group showed normal liver with only mild staining of sinusoids with no staining (negative) in hepatocytes (**B**) BDL group showed strong cytoplasmic staining of the hepatocytes (**C**) Diacerein-treated group (10 mg/kg/day) showed moderate cytoplasmic staining of the hepatocytes (**D**) Diacerein-treated group (30 mg/kg/day) showed only focal weak cytoplasmic staining of the hepatocytes (**E**) Diacerein-treated group (50 mg/kg/day) showed only mild staining of sinusoids with no staining (negative) in hepatocytes.

Furthermore, the immunohistochemical analysis of CHOP showed a highly significant (p < 0.001) increase in the expression of this protein in the liver samples of the BDL group (37.5% *moderate*, 62.5% *strong*) as compared to the sham-operated rats. Oral treatment with diacerein for four weeks significantly reduced this BDL-induced enhancement of CHOP immunoreactivity in a dose-dependent manner as follows: diacerein-treated group 10 mg/kg/day (12.5% *negative*, 37.5% *weak*, 50% *moderate*), diacerein-treated group 30 mg/kg/day (25% *negative*, 50% *weak*, 25% *moderate*) and diacerein-treated group 50 mg/kg/day (50% *negative*, 37.5% *weak*, 12.5% *moderate*) (Table 1, Fig. 5).

**Table 1:** Effect of different doses of diacerein on CHOP immunoreactivity induced by BDL in different experimental groups.

Group	Sham-operated group		BDL group		BDL + diacerein (10 mg/kg)		BDL + diacerein (30 mg/kg)		BDL + diacerein (50 mg/kg)		Chi square (X^2^)	p value	p value
	N	%	N	%	N	%	N	%	N	%			
CHOP immune-reactivity													
Negative (0)	8	100	0	0	0	12.5	1	25	4	50	26.895	0.000**	0.000**
Weak (1)	0	0	0	0	2	37.5	4	50	3	37.5	9.176	0.057	
Moderate (2)	0	0	3	37.5	6	50	3	25	1	12.5	12.080	0.017	
Strong (3)	0	0	5	62.5	0	0	0	0	0	0	22.857	0.000**	

*Statistically significant difference (p ≤ 0.01), **Statistically highly significant difference (p ≤ 0.001), n: number (number of sacrificed rats in each group = 8 rats), %: Percent. There were statistically significant differences between all groups regarding negative (0) and strong (3) CHOP immunoreactivity.

### Effect of diacerein on hepatic p-JNK protein level

The p-JNK level was significantly (p < 0.05) up-regulated in the BDL group as compared to the sham-operated group. A significant (p < 0.05) down-regulation in the level of p-JNK was observed in diacerein-treated groups as compared to the BDL group. The effect of diacerein on p-JNK level was dose-dependent (Fig. 4D).

### Effect of diacerein on hepatic protein contents of TGF˗β1, α-SMA, COL-1 and hydroxyproline

**Figure 6:**
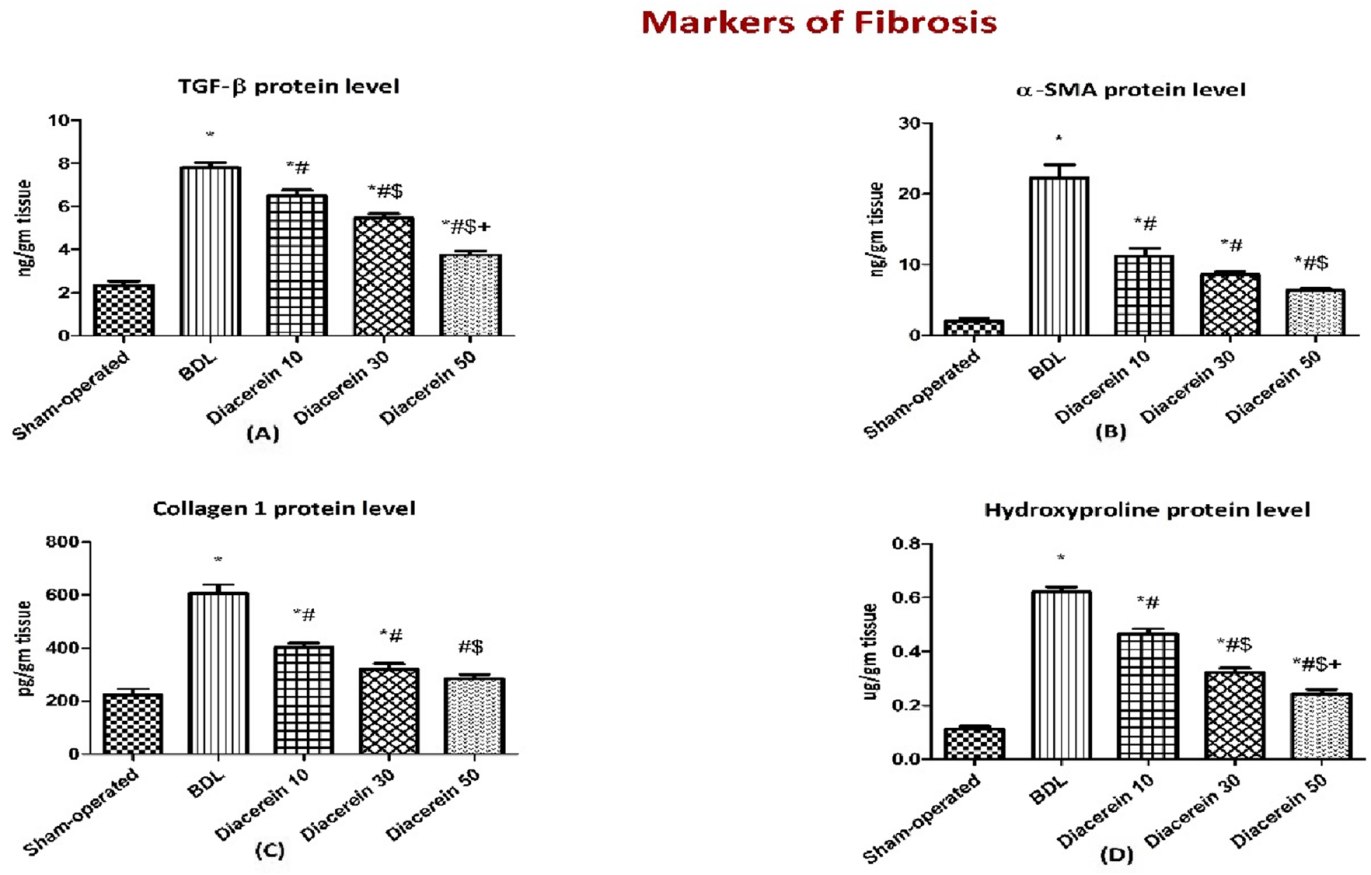
Bar charts showing the effect of oral administration of different doses of diacerein on the hepatic levels of the fibrotic markers: (**A**) TGF-β1 (ng/gm tissue), (**B**) α-SMA (ng/gm tissue), (**C**) collagen 1 (pg/gm tissue), and (**D**) hydroxyproline *(ug/gm tissue)* content in different experimental groups. The values of different groups were presented as means ± SE (n = 8 rats per group). *p < 0.05 versus the sham-operated group; ^#^p < 0.05 versus the BDL group; ^$^p < 0.05 versus the diacerein-treated group (10 mg/kg/day); ^+^p < 0.05 versus the diacerein-treated group (30 mg/kg/day).

The BDL rats showed significantly (p < 0.05) increased hepatic protein levels of the profibrogenic mediators, including TGF˗β1, α-SMA, COL-1, and hydroxyproline as compared to the sham-operated group. Administration of diacerein to rats subjected to BDL showed a significant (p < 0.05) reduction in all these parameters in a dose-dependent manner versus the BDL group (Fig. 6A–D).

### Effect of diacerein on the hepatic protein contents of caspase-3 and BAX

**Figure 7:**
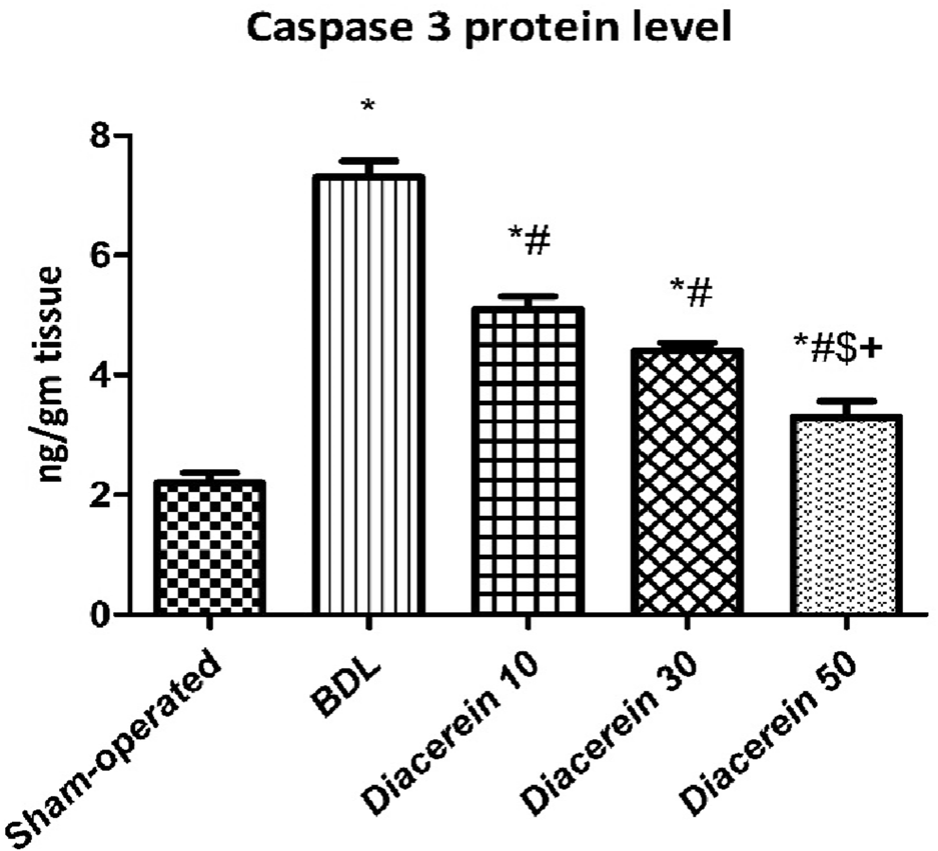
Bar charts showing the effect of oral administration of different doses of diacerein on hepatic levels of caspase 3 protein content (ng/gm tissue) in different experimental groups. The values of different groups were presented as means ± SE (n = 8 rats per group). *p < 0.05 versus the sham-operated group; ^#^p < 0.05 versus the BDL group; ^$^p < 0.05 versus the diacerein-treated group (10 mg/kg/day); ^+^p < 0.05 versus the diacerein-treated group (30 mg/kg/day).

The hepatic concentration of caspase 3 was significantly (p < 0.05) increased in the BDL group as compared to the sham-operated group. Treatment with diacerein (10, 30, and 50 mg/kg/day) significantly (p < 0.05) decreased caspase 3 level in a dose-dependent manner as compared to the BDL rats (Fig. 7).

**Figure 8:**
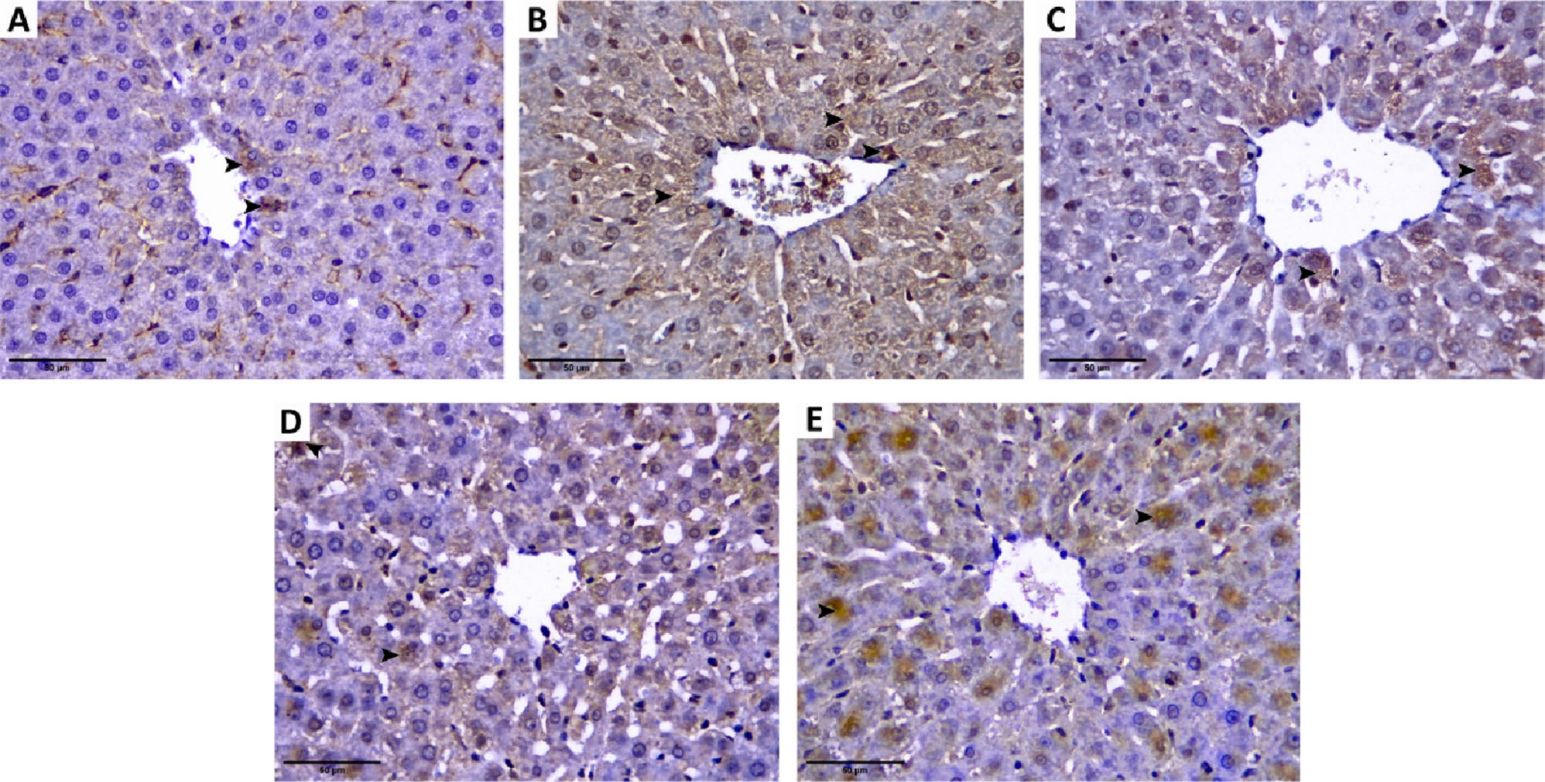
Immunohistochemistry staining images (× 400) showing hepatic expression of BAX protein. Positively stained cytoplasm is taking brown colour (arrowhead). (**A**) Sham-operated group showing very weak cytoplasmic staining, including few hepatocytes; (**B**) BDL group showing strong positive cytoplasmic staining of the hepatocytes; (**C**) and (**D**) Diacerein-treated groups (10 mg/kg/day and 30 mg/kg/day) showing moderate scattered cytoplasmic staining of the hepatocytes; (**E**) Diacerein-treated group (50 mg/kg/day) showing weak hepatic cytoplasmic staining.

Regarding BAX immunostaining, the sham-operated rats showed very weak cytoplasmic staining, while the BDL rats showed strong positive cytoplasmic staining of the hepatocytes. BAX immunostaining decreased with diacerein, with the best effect with diacerein (50 mg/kg/day) (Fig. 8).

### Effect of diacerein on histopathological changes

**Figure 9:**
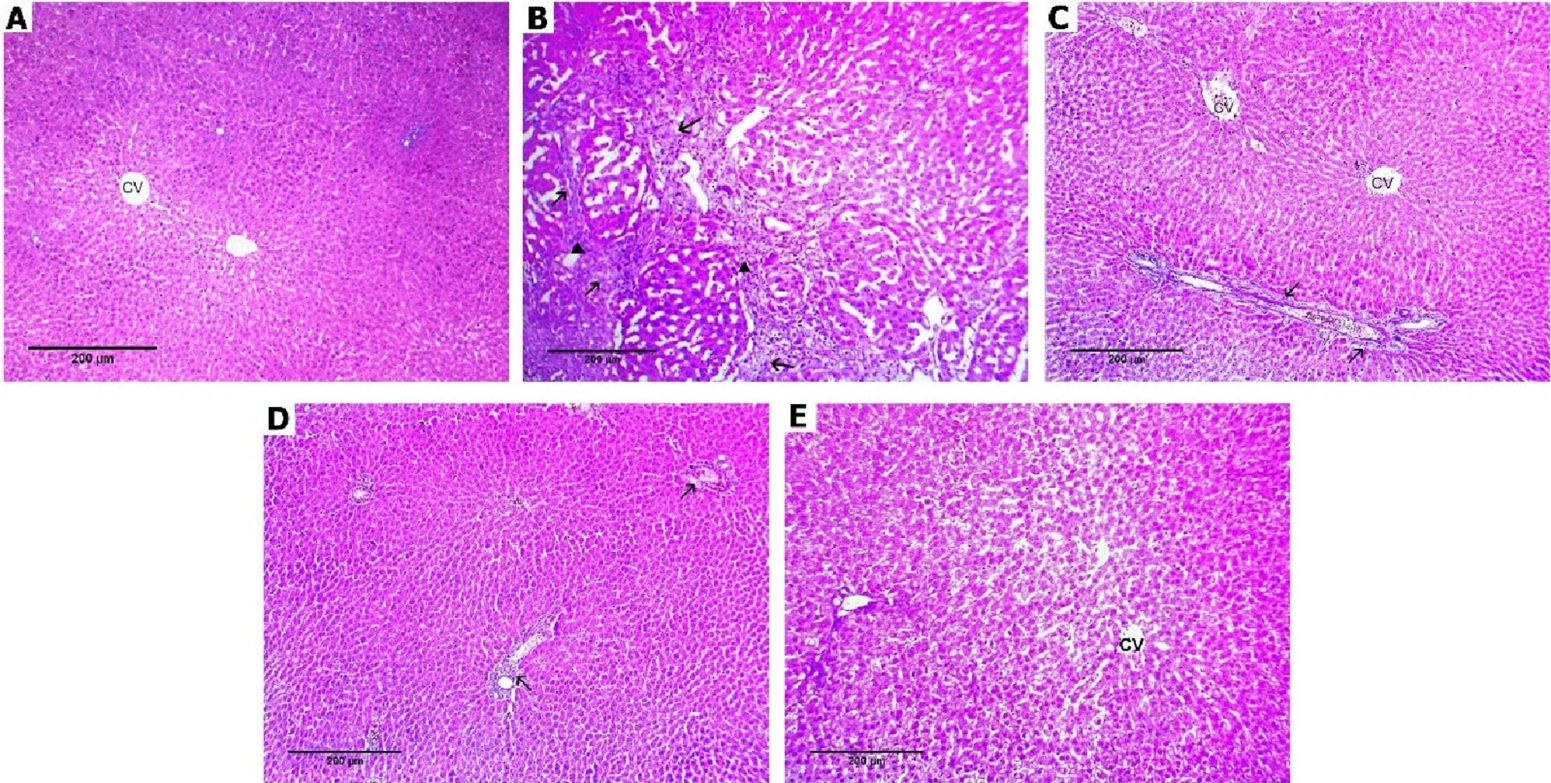
Photomicrographs of liver tissue stained with hematoxylin and eosin × 100 (**A**) Sham-operated group showing preserved hepatocytes’ arrangement with no inflammation or fibrosis (grade 0), (**B**) BDL group showing marked fibrosis (arrows) with moderate inflammation (arrowheads) (grade 3), (**C**) Diacerein-treated group (10 mg/kg/day) showing portal fibrosis (arrows) with bile duct proliferation (asterisks), mild lobular inflammation (arrowheads) (grade 2), (**D**) Diacerein-treated group (30 mg/kg/day) showing portal fibrosis (arrow) and mild lobular and portal inflammation (arrowheads) with occasional hepatocyte necrosis (dashed arrows) **(**grade 2), (**E**) Diacerein-treated group (50 mg/kg/day) showing dilated sinusoids in a preserved architecture with occasional necrosis (dashed arrows), minimal portal inflammation (arrowhead) and vacuolar degeneration (broken arrows) (grade 1).

*Regarding inflammatory changes*, histopathological examination of H&E-stained liver sections of the sham-operated group revealed regular hepatic architecture with normal-appearing hepatocytes with no inflammation. On the contrary, BDL rats showed moderate portal inflammation with hepatic architectural distortion (25% *grade 2*, 75% *grade 3*). Liver sections of rats treated with diacerein at the above-selected doses showed a decrease in the severity of histopathological changes with a highly significant (p < 0.001) improvement in the hepatic grade of inflammation as compared to the BDL group as follows: diacerein-treated group 10 mg/kg/day (12.5% *grade 0*, 50% *grade 1*, 37.5% *grade 2*), diacerein-treated group 30 mg/kg/day (25% *grade 0*, 50% *grade 1*, 25% *grade 2*), and diacerein-treated group 50 mg/kg/day (25% *grade 0*, 62.5% *grade 1*, 12.5% *grade 2*). Interestingly, amelioration of inflammation appeared to be greatest in the group treated with diacerein at 50 mg/kg/day (Table 2, Fig. 9).

**Table 2:** Effect of different doses of diacerein on inflammatory changes induced by BDL in different experimental groups.

Group	Sham-operated group		BDL group		BDL + diacerein (10 mg/kg)		BDL + diacerein (30 mg/kg)		BDL + diacerein (50 mg/kg)		Chi square (X^2^)	p value	p value
	N	%	N	%	N	%	N	%	N	%			
Grade of inflammation													
Grade (0)	8	100	0	0	1	12.5	2	25	2	25	22.336	0.000**	0.000**
Grade (1)	0	0	0	0	4	50	4	50	5	62.5	13.219	0.010*	
Grade (2)	0	0	2	25	3	37.5	2	25	1	12.5	4.063	0.398	
Grade (3)	0	0	6	75	0	0	0	0	0	0	28.235	0.000**	
Grade (4)	0	0	0	0	0	0	0	0	0	0	0	0	

*Statistically significant difference (p ≤ 0.01), **Statistically highly significant difference (p ≤ 0.001), n: number (number of sacrificed rats in each group = 8 rats), %: Percent. There are statistically significant differences between all groups regarding all grades of inflammatory changes except grade 2.

**Figure 10:**
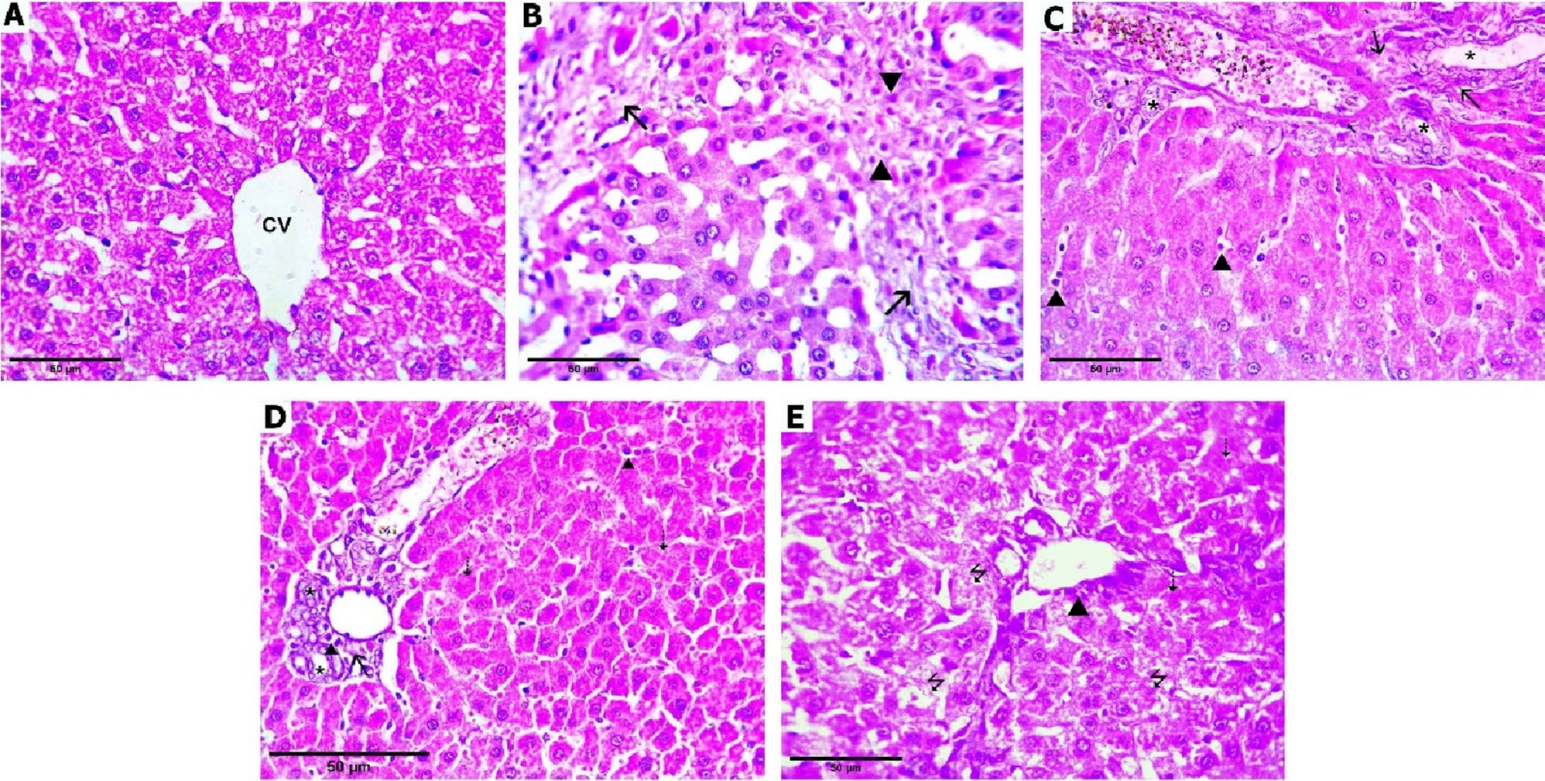
Photomicrographs of liver tissue stained with hematoxylin and eosin (H&E × 400). (**A**) Sham-operated group showing preserved hepatocytes’ arrangement with no fibrosis (F0); (**B**) BDL group showing marked septal fibrosis (arrows) and moderate inflammation (arrowheads) with the formation of regenerative (cirrhotic) nodules (F4); (**C**) Diacerein-treated group (10 mg/kg/day) showing few portal-portal fibrosis (arrows) (F2); (**D**) Diacerein-treated group (30 mg/kg/day) showing portal fibrosis (arrows) with the absence of normal central veins distribution (F1); (**E**) Diacerein-treated group (50 mg/kg/day) showing dilated sinusoids in a preserved architecture with no fibrosis (F0).

**Figure 11:**
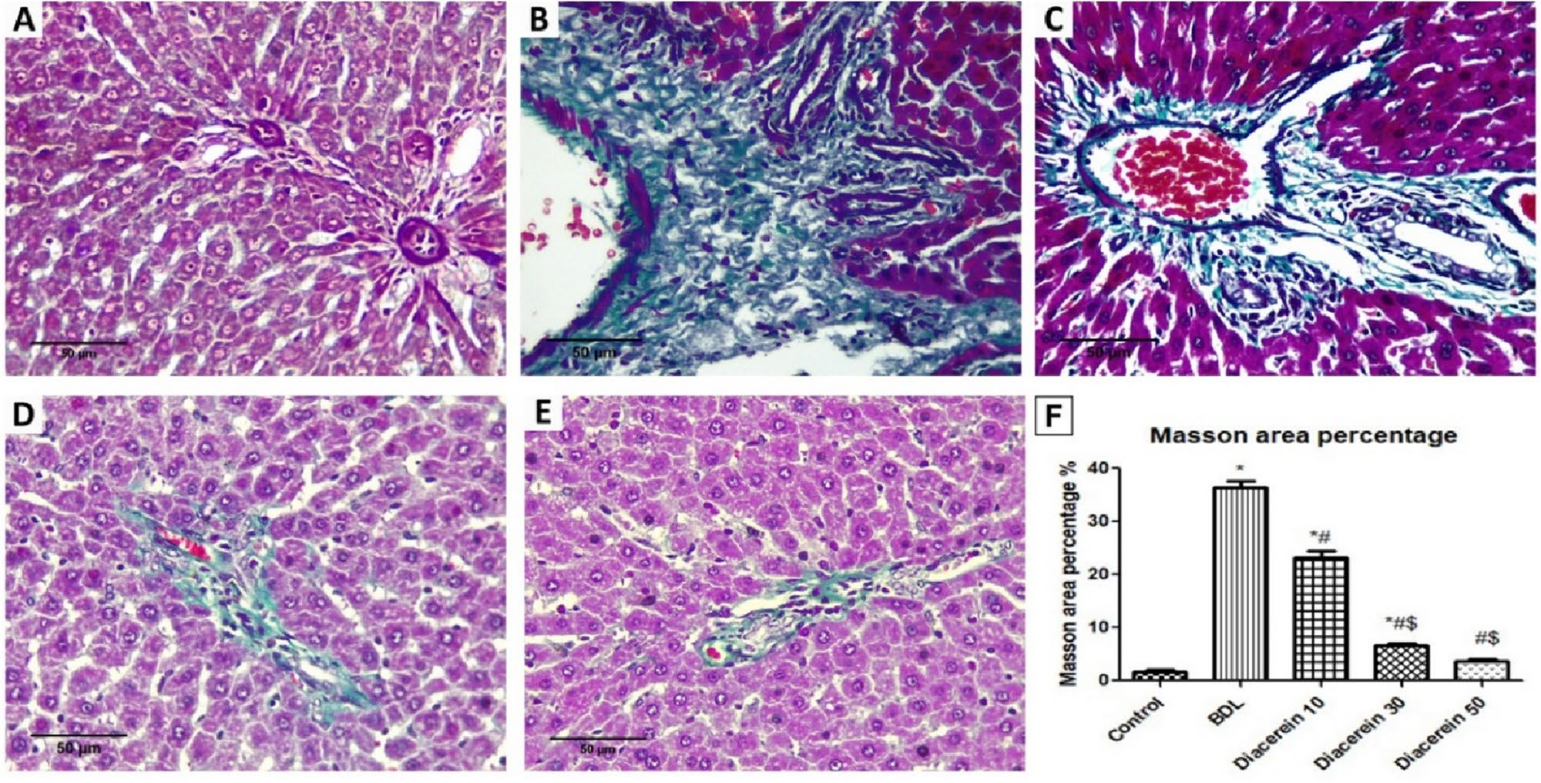
Photomicrographs of liver tissue stained with Masson trichrome for detection of collagen fibers (green colour) (H&E × 400); (**A**) Sham-operated group showing no fibrosis or collagen deposition (F0); (**B**) BDL group showing marked fibrosis and cirrhosis (F4); (**C**) Diacerein-treated group (10 mg/kg/day) showing periportal fibrosis with septa and collagen deposition (F2); (**D**) Diacerein-treated group (30 mg/kg/day) showing portal fibrosis with few septa and collagen deposition (F2); (**E**) Diacerein-treated group (50 mg/kg/day) showing minimal amount of fibrosis without septa (F1). (**F**) Morphometrical analysis showing the area percentage of collagen fibers in Masson’s hepatic sections in the studied groups.

*Regarding fibrotic changes*, histopathological examination of H&E-stained liver sections of the sham-operated group revealed regular hepatic architecture without fibrosis. On the other hand, liver sections from BDL rats showed marked septal fibrosis with the formation of regenerative (cirrhotic) nodules with hepatic architectural distortion (12.5% *stage 2*, 37.5% *stage 3*, 50% *stage 4*). The liver section of rats treated with diacerein showed a decrease in the severity of fibrotic changes with significant (p < 0.001) improvement in the stage of hepatic fibrosis compared to the BDL group as follows: diacerein-treated group 10 mg/kg/day (12.5% *stage 1*, 62.5% *stage 2*, 25% *stage 3*), diacerein-treated group 30 mg/kg/day (12.5% *stage 0*, 37.5% *stage 1*, 50% *stage 2*), and diacerein-treated group 50 mg/kg/day (12.5% *stage 0*, 75% *stage 1*, 12.5% *stage 2*). The amelioration of hepatic fibrotic change was the best in the group treated with diacerein at 50 mg/kg/day (Table 3; Figs. 10 and 11).

**Table 3:** Effect of different doses of diacerein on fibrotic changes induced by BDL in different experimental groups.

Group	Sham-operated group		BDL group		BDL + diacerein (10 mg/kg)		BDL + diacerein (30 mg/kg)		BDL + diacerein (50 mg/kg)		Chi square (X^2^)	p value	p value
	N	%	N	%	N	%	N	%	N	%			
Stage of fibrosis													
Stage (0)	7	87.5	0	0	0	0	1	12.5	1	12.5	24.946	0.000**	0.000**
Stage (1)	1	12.5	0	0	1	12.5	3	37.5	6	75	14.295	0.006*	
Stage (2)	0	0	1	12.5	5	62.5	4	50	1	12.5	11.787	0.019	
Stage (3)	0	0	3	37.5	2	25	0	0	0	0	9.143	0.058	
Stage (4)	0	0	4	50	0	0	0	0	0	0	17.778	0.001**	

Comparison of fibrotic stages among different experimental groups *Statistically significant difference (p ≤ 0.01), **Statistically highly significant difference (p ≤ 0.001), n: number (number of sacrificed rats in each group = 8 rats), %: Percent. There are statistically significant differences between all groups regarding all stages of fibrotic changes except stages 2 and 3.

## Discussion

Cholestatic liver disorders are chronic diseases initiated by bile flow and/or secretion impairment, which in turn leads to the accumulation of toxic hydrophobic bile acids within the liver, inducing severe liver damage with later deterioration to fibrosis, cirrhosis, and liver failure^32^.

Obstructive cholestasis induced by BDL in this work is a frequently used model in animals because it mimics biliary obstruction occurring in humans, allowing us to explore the pathogenesis of this disorder^3^.

Unfortunately, there are presently no available therapy options for this disorder, making it necessary to investigate the molecular mechanisms of cholestasis induced liver fibrosis and to develop a new approach for inhibiting or slowing the progression of the fibrogenic process. Therefore, the goal of the current study is to evaluate the effect of diacerein in rats with BDL-induced liver fibrosis and elucidate the underlying mechanisms.

In this work, cholestasis induced by BDL caused liver damage as evidenced by elevations in serum levels of hepatic enzymes (AST, ALT, and ALP) and direct bilirubin (DB). Moreover, histopathological changes further confirmed the BDL-induced liver injury. These findings are in line with the study by Gheitasi et al.^33^. An increase in these enzymes reflects the deterioration of structural integrity and permeability of hepatocytes membrane by the toxic bile salts' detergent effect, allowing these enzymes to escape into the blood^34^. Moreover, the increased direct bilirubin serum level appears to be caused by an increase in the concentration gradient between the plasma and liver cells resulting from bile duct obstruction^35^. Diacerein administration in this study showed a hepatoprotective effect in a dose-dependent manner, as proved by improved liver function parameters (AST, ALT, ALP, and DB), as well as hepatic architecture detected histologically, suggesting the ability of diacerein to maintain the structure of hepatocytes membrane. These results were consistent with those reported by Ibrahim et al.^36^.

In our study, BDL group exhibited an increase in the lipid peroxidation end product, MDA, coupled with a reduction in SOD activity in hepatic tissue. Similar results were reported by Barmoudeh et al.^37^. Oxidative stress is a prominent phenomenon in cholestasis that develops from an imbalance between oxidants and antioxidants^38^. Free radicals are generated when potentially hazardous bile acids are retained, which then promote lipid peroxidation and the generation of MDA. Additionally, excessive free radicals also cause a discernible decline in SOD, a crucial antioxidant enzyme^39^. In this work, diacerein-treated rats restored the antioxidant system, as indicated by the reduction in hepatic MDA level and the increase in hepatic SOD activity. This antioxidant effect of diacerein might be attributed to its anthraquinone structure and anti-inflammatory properties. In line with these results, Tamura et al.^40^ illustrated that diacerein could suppress free radical production in gastric ulceration induced by indomethacin based on its anti-inflammatory effect on neutrophils. Also, Refaie and El-Hussieny^41^ reported the antioxidant impact of diacerein in endometrial hyperplasia and atypia models induced by estradiol benzoate.

In this experiment, BDL rats showed up-regulation of hepatic protein and gene expression of HMGB1 and gene expression of RAGE, which is in harmony with the study by Wen et al.^42^. These findings suggest that the HMGB1/RAGE pathway may contribute to the molecular mechanisms of cholestasis-induced liver fibrosis. Inflammation is the cornerstone factor in the pathogenesis of this disorder^43^. The inflammatory cytokine HMGB1 is expelled outside the nucleus into the cytoplasm either *passively* from damaged hepatocyte cells or *actively* from inflammatory cells^7^, where it interacts with its cell surface receptors, particularly RAGE, which is abundantly expressed on different types of liver cells^12^. Emerging studies reported that HMGB1 is involved in the fibrosis of different organs^9–11^. Intriguingly, HMGB1 neutralizing Ab attenuated fibrosis of liver induced by CCl_4_ in mice^44^, proving the value of HMGB1 in the pathophysiology of hepatic fibrogenesis and demonstrating the significance of using its inhibition to treat liver fibrosis. It appears that the fibrotic signal of HMGB1 has been mediated via RAGE, as reported by Ge et al.^44^ who found, in an in vitro study, that HSCs migration induced by HMGB1 was mediated by increasing RAGE expression. In a rat lung injury induced by arsenic, Wang et al.^13^ demonstrated that HMGB1 mediates lung inflammation and fibrosis through RAGE. Our study revealed that diacerein reduced hepatic protein and gene expression of HMGB1 and gene expression of RAGE. These findings are in accordance with a recent study by Kamel et al.^45^ who proved that diacerein's hepatoprotective impact on acetaminophen-induced hepatotoxicity was brought about by inhibition of the HMGB1/TLR4 pathway.

Rats who underwent BDL in this study demonstrated elevated hepatic NF-κB and IL˗1β levels. This was further confirmed histologically by the detection of remarkable inflammatory changes with marked fibrosis, which may be brought on by HMGB1/RAGE overexpression. Similar findings were obtained by Pan et al.^46^. NF-κB is a pleiotropic transcription factor that plays a fundamental role in fibrogenesis by inducing the expression of numerous pro-inflammatory^47^, as well as pro-fibrogenic cytokines^48^. One of the pro-inflammatory cytokines transcribed by NF-κB is IL-1β, which induces HSCs activation^49^. Stimulation of RAGE has been implicated in the activation of NF-κB as confirmed by Cai et al.^50^ who showed that hepatic fibrosis brought on by CCl_4_ was reduced following RAGE gene silencing, and this beneficial effect was achieved by inhibiting NF-κB. In this work, diacerein treatment attenuated the increase in hepatic NF-κB, and IL˗1β levels induced by BDL. An improvement of the inflammatory grade in liver sections of rats that received diacerein further confirmed its anti-inflammatory effects. These results are in accordance with preceding studies^36,51^.

In this work, rats subjected to BDL showed up-regulation of hepatic GRP78, IRE1α, and PERK mRNA expression and p-JNK level, as well as enhancement of CHOP immunoreactivity, pointing to the enhancement of ER stress in this disorder, which may be linked to HMGB1/RAGE up-regulation. These findings are consistent with prior studies^52,53^. A heat shock protein, GRP78, interacts with generated unfolded proteins, followed by activating ER stress sensors^54^. CHOP is the primary apoptotic molecule that is up-regulated in response to PERK^55^. Tamaki et al.^56^ found that hepatic apoptosis and fibrosis were reduced in mice lacking CHOP, confirming its significance in the pathogenesis of this disease. Activation of another ER stress sensor, IRE1α, triggers downstream factors, JNK and NF-κB^57^. In liver tissue, c-Jun N-terminal kinase is a distinctive effector of mitogen-activated protein kinase (MAPK)^58^. Accumulating evidence has reported that JNK is implicated in fibrosis by triggering several genes linked to inflammation and apoptosis^59^. Most importantly, some preceding studies have found that HMGB1 is the upstream regulator of ER stress; Huang et al.^18^ and He et al.^19^ demonstrated that HMGB1/RAGE interaction induced endothelial cell apoptosis and acute respiratory distress syndrome in patients and animals through activation of ER stress, respectively. In support of this observation, Lai et al.^60^ revealed that ER stress was attenuated by HMGB1 inhibition or neutralization in acute renal injury brought on by intestinal ischemia/reperfusion. In our study, treatment of BDL rats with diacerein mitigated the activation of ER stress markers (hepatic GRP78, IRE1α, PERK, p-JKN, and CHOP). These results are in harmony with the results of Tobar et al.^61^ and da Silva et al.^62^.

In our study, BDL group showed an elevation in hepatic fibrogenic parameters [TGF-β1, α-SMA, collagen I, and hydroxyproline] together with marked collagen deposition and fibrosis upon histopathological evaluation, which are consistent with the results of Moslemi et al.^34^. TGF-β1, a pro-fibrogenic cytokine, is intimately connected to the fibrogenesis process via encouraging HSCs activation, collagen expression, and preventing its degradation^48^. One of the most crucial fibrogenic indices, which reflect HSCs activation, is *α*-SMA^63^. Myofibroblasts are formed after activation of HSCs, generating considerable amounts of ECM, particularly collagen 1^5^, whose structural integrity depends on the presence of hydroxyproline, a crucial collagen component^64^. Wu et al.^65^ reported that TGF-β1 expression in HSCs could be promoted via activated JNK. Furthermore, Wen et al.^42^ demonstrated that activated MAPKs, including JNK, triggered collagen expression in HSCs in fibrotic rat livers induced by CCL_4_. More importantly, the same study found that MAPKs' fibrotic effects were mediated through the HMGB/RAGE pathway. Diacerein-treated rats in this experiment revealed an attenuation in the levels of these fibrogenic molecules along with the improvement of fibrotic alterations in liver sections of BDL rats, suggesting the ability of diacerein to decrease HSCs activation. These data were in line with those of Torina et al.^66^ and Barakat et al.^67^ who proved the anti-fibrotic effect of diacerein in rats with myocardial infarction and cisplatin-induced renal injury, respectively.

In our work, an elevation of caspase 3 and BAX immunostaining was observed in the liver of BDL rats, which is in accordance with those obtained by Nasehi et al.^68^. Caspase 3, a primary protease enzyme, is frequently used to detect apoptosis^69^. Removing generated apoptotic bodies through phagocytosis triggers the release of cytokines and TGF-β1, which activate HSCs and ultimately aggravate fibrogenic cascades^70^. Diacerein administration to BDL rats in our study showed a reduction in hepatic levels of caspase 3 and BAX immunostaining, indicating an anti-apoptotic effect of diacerein in cholestatic liver fibrosis, which comes with other data reported by Elshal and Abdelmageed^71^ and Bu et al.^72^.

## Conclusion

This study revealed for the first time that diacerein treatment, in a dose-dependent manner, had a hepatoprotective effect on experimental liver fibrosis induced by BDL. This effect may be linked to Modulation HMGB1/RAGE/NF-κB/JNK and ER stress signalling pathways. We hope that these findings can open the way for new strategies in the treatment of cholestatic liver disorders.

## Data Availability

The used and analysed datasets in our study are available from the corresponding author on reasonable request.

## Abbreviations

ECM: Extracellular matrix proteins
DAMPs: Damage-associated molecular patterns
HMGB1: High mobility group box 1
RAGE: Advanced glycation end products receptor
ER: Endoplasmic reticulum
IRE1α: Inositol-requiring enzyme 1
ATF6: Activating transcription factor 6
PERK: PKR-like ER protein kinase
IL-1β: Interleukin-1 beta
NF-κB: Nuclear factor-κB
CMC-Na: Sodium carboxymethyl cellulose
BDL: Bile duct ligation
i.p: Intraperitoneal injection
ALT: Alanine aminotransferase
AST: Aspartate aminotransferase
ALP: Alkaline phosphatase
DB: Direct bilirubin
MDA: Malondialdehyde
SOD: Superoxide dismutase
TGF˗β1: Transforming growth factor β1
p-JNK: Phosphorylated c-Jun N-terminal kinase
COL-1: Collagen-1
α-SMA: α-Smooth muscle actin
GRP78: Glucose-regulated protein 78
H&E: Hematoxylin and eosin
CHOP: CCAAT/enhancer-binding protein homologous protein
ANOVA: One way analysis of variance
